# Evaluation of *Salmonella* presence in selected United States feed mills

**DOI:** 10.1002/mbo3.711

**Published:** 2018-08-29

**Authors:** Gabriela Magossi, Natalia Cernicchiaro, Steve Dritz, Terry Houser, Jason Woodworth, Cassandra Jones, Valentina Trinetta

**Affiliations:** ^1^ Food Science Institute Kansas State University Manhattan Kansas; ^2^ Diagnostic Medicine/Pathobiology College of Veterinary Medicine Kansas State University Manhattan Kansas; ^3^ Department of Animal Sciences and Industry Kansas State University Manhattan Kansas

**Keywords:** contamination, feed, feed mills, microbial entry route, *Salmonella*

## Abstract

*Salmonella* is a pathogen of public health concern. Each year, *Salmonella* infections cost to the food industry approximately $2.3 billion and 33% of the reported cases are associated with beef, poultry, or pork. Pathogen presence in feed mills can represent one of the many potential routes for entry and transmission into the food production chain. Nevertheless, little is known about *Salmonella* incidence and association with these types of environments. The objective of this study was to investigate *Salmonella* presence in different feed mills across the United States. Eleven facilities were selected in eight states and 12 sites were sampled within each feed mill. Samples were analyzed following the FSIS guidelines for isolation and identification of *Salmonella*. Positive isolates were further investigated by a PCR analysis targeting the *invA* gene to differentiate for *Salmonella enterica*. The total number of environmental samples collected was 237: 66% resulted culture positive and 13.1% were PCR positive. All sampled feed mills had at least one culture positive site and following production flow the number of positive samples decreased from ingredient receiving to final product. These preliminary results demonstrate the presence of *Salmonella* in selected United States feed mills and suggest their potential role as vehicle for pathogen transmission and spread into the food production chain.

## 1 INTRODUCTION

*Salmonella* is a resilient microorganism that can live in low water activity conditions and adapt to different temperatures (Podolack, Enache, Stone, Black, & Elliott, 2010). This pathogen can survive outside the animal host and in the environment for long periods of time (Baer, Miller, & Dilger, 2013 *)*. The Centers for Disease Control and Prevention (CDC) estimated that *Salmonella* is responsible for approximately one million foodborne illnesses, 19,000 hospitalizations and 380 deaths in the USA each year (CDC, 2014). The yearly impact for the food industry is around $2.3 billion. From 2006 to 2015, the number of cases of *Salmonella* linked to pork products has increased (CDC, 2014). Although pork has the lowest association with human foodborne illness, when compared to beef and chicken it is the most consumed meat in the world (Delgado, Rosegrant, Steinfeld, Ehui, & Courbois, 2001). Therefore, *Salmonella* has become a food safety concern also for the American swine industry: ensuring the safety of pork is essential for producers to maintain animal and human health, and to continue serving export markets (Baer et al., 2013 *)*. Several studies have estimated the presence of *Salmonella* in feed as generally low, and historically no evidence of direct link to animal or human illness has been demonstrated in US (Burns et al., 2015; Cochrane et al., 2015; Davies, Hurd, Funk, Fedorka‐Cray, & Jones, 2004; Molla et al., 2010). Nevertheless, the importance of feed as pathogen contamination source in pigs, the potential risk of transmission and survival in slaughter houses and the possible infection for consumers has been highlighted as significant and potentially high in several risk assessment models *(*Rönnqvist, Välttilä, Ranta, & Tuominen, 2017; Österberg, Vågsholm, Boqvist, & Sternberg Lewerin, 2006). Surveillance programs for *Salmonella* in animal products and feed have been already implemented in USA (Animal Feed Safety System, Feed Contaminants Program from 2002 to 2006, and the *Salmonella* Assignment from 2007 to 2009) and in Europe (Swedish National *Salmonella* Control Programme) (Abrahantes, Bollaerts, Aerts, Ogunsanya, & Van der Stede, 2009; Österberg et al., 2006). Moreover, a surveillance study conducted in USA from 2002 to 2009 reported that 12.5% of feed and feed ingredient samples collected from manufacturing facilities were contaminated with *Salmonella* (Li et al., 2012). These results support the importance to investigate pathogen presence and possible infection sources from feed to fork. The risk of salmonellosis from feed is difficult to quantify due to inconsistent data, sampling constrains, and lack of epidemiological information (Crump & Griffin, 2002; Jones, 2011). Limited practices have been implemented for animal feed environments, even if these facilities have been recognized as potential source of infections in different occasions (Podolack et al., 2010; Rostagno & Callaway, 2012). Therefore, the objectives of this study were to: (a) evaluate the presence of *Salmonella* in selected United States commercial animal feed mills; and (b) preliminary characterize the prevalence of the pathogen in relation to sampling site and processing‐associated risk factors.

## 2 MATERIALS AND METHODS

### 2.1 Swabbing method and sites

**Figure 1:**
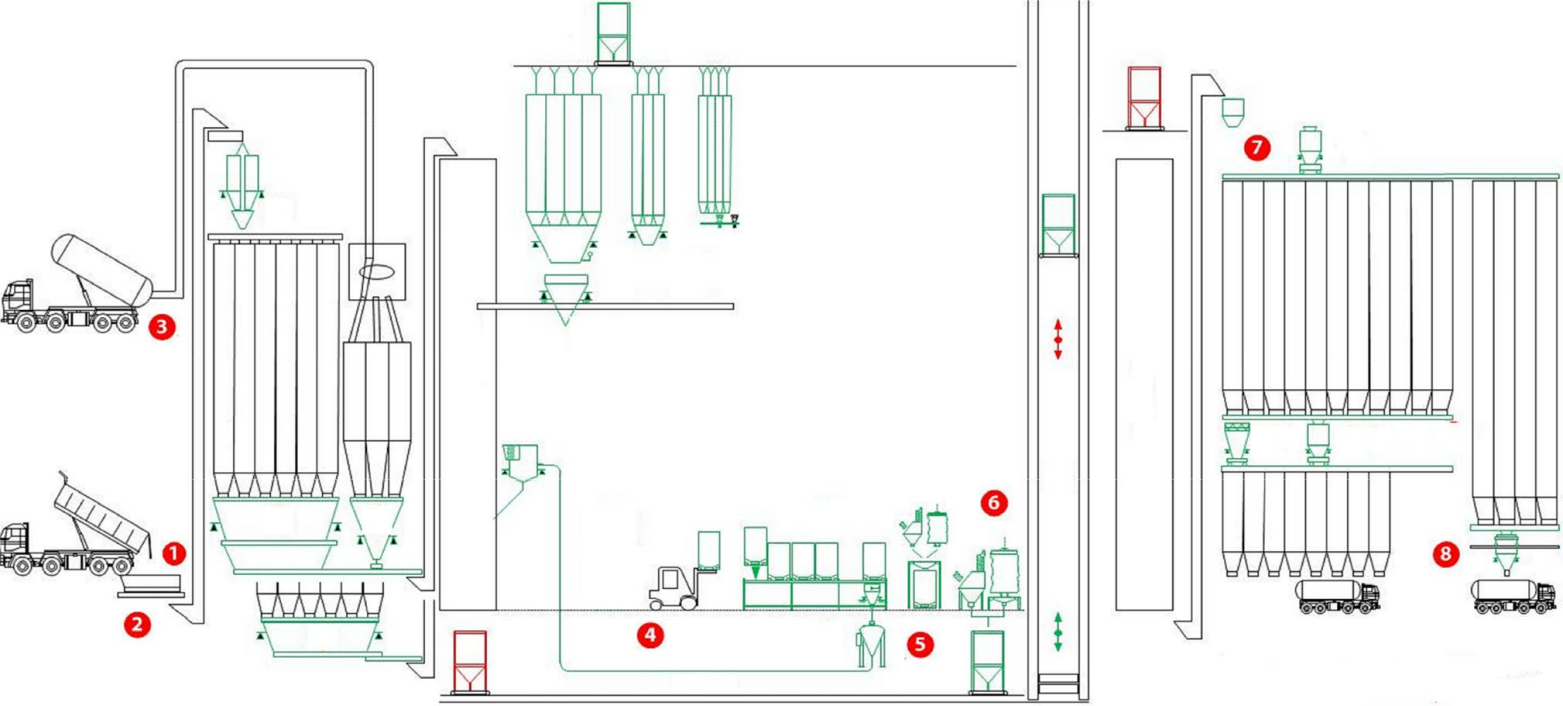
General layout of a feed mill production system with highlighted sampling sites (adapted from http://www.kse.nl/en/alfra/). 1. Receiving ingredients pit gratin. 2. Receiving area. 3. Fat intake inlet. 4. Warehouse area. 5. Manufacturing area. 6. Pellet mill. 7. Load‐out auger. 8. Discharge bin boot

A diverse geographical pool of 11 feed manufacturing facilities, representative of the US swine production areas, were selected for this study. One location was identified in Colorado, Illinois, Indiana, Minnesota, and Oklahoma, while two in Iowa, Kansas, and North Carolina. Six mills produced only mash feed, while the other five facilities produced both mash and pelleted feed with average conditioning temperatures of 71°C for 45 s. Mills were sampled once between the months of October and November 2016. Twelve sites within each facility were targeted for a total of 237 samples. The sites were selected considering production flow, people traffic and dust accumulation (Table 1). Samples were collected with a sterile sponge‐stick presoaked in 10 ml of Buffered Peptone Water (3M, St Paul, MN) using a 10 × 10 cm sterile template. Surfaces in receiving ingredient pit grating, floors in receiving area, manufacturing area, warehouse and control/brake room were sampled in triplicates. Single samples were collected from fat intake inlet, exterior of pellet mill, finished product bin boot/product discharge, load‐out auger and broom. Worker shoes samples were collected from both left and right shoe. Finished feed was obtained from fresh feed manufactured the same day of sample collection, usually after conditioning (Figure 1). Only for feed samples the method described in Chapter 5 of the Bacteriological Analytical Manual was followed and a 50 g feed portion was used for further testing (Bacterial Analytical Manual, 2011). All samples were kept under refrigeration conditions and transported to the laboratory. Processing and testing of samples was conducted within 48 hr of sampling.

**Table 1:** Presence of *Salmonella* culture positive (C+) and PCR positive (**PCR+**) samples in feed mill facilities selected in this study

	Pelleted feel mills^a^						Mash feed mills^a^					C+ (%)	PCR+ (%)
	1	2	3	4	5	6	7	8	9	10	11		
Sampling site within the production flow													
Ingredient pit grating^b^	+++	−−+	**++**−	++**+**	+++	++**+**	+	+++	−+−	**+**++	+−+	80.6	16.1
Floor dust in receiving^b^	+−−	−−−	+++	++**+**	**++**+	**+**++	+	+++	+−+	++**+**	+++	80.6	16.1
Floor dust in manufacturing area^b^	**+**−−	−−−	−+−	+**++**	−−−	**+**+**+**	**+**	+++	++−	+++	−++	61.3	19.4
Floor dust in break or control room^b^	−**+**−	−++	+−−	**+++**	−++	+−−	**+**	+++	−−−	+++	+++	64.5	16.1
Floor dust in warehouse^c^	−−−	+−+	−−−	**+**+−	−++	++−	−	−+−	−++	+++	+++	54.8	3.2
Exterior of pellet mill	−	−	−	**+**	−	+	n/a	n/a	n/a	n/a	n/a	33.3	16.7
Finished product bin boot	−	+	+	+	+	**+**	**+**	+	−	+	+	81.8	18.2
Load−out auger	−	−	−	−	+	+	+	+	−	+	−	45.5	0.0
Finished feed	−	−	−	**+**	−	+	+	+	−	+	−	41.7	8.8
Sampling site outside the production flow													
Worker shoes^c^	++	−+	++	+**+**	++	+**+**	+	++	++	++	++	95.2	9.5
Broom	−	**+**	−	+	−	**+**	+	+	−	**+**	+	63.6	27.3
Fat intake inlet	−	−	−	d	−	+	−	−	−	+	−	20.0	0.0
Total %												62.2	19.8

*Note*.

The % of PP and CP at the end of each row were calucalted for sample sites.

n/a, site not present in mash facilities.

^a^Mills name and location were substituted by number to protect collaborators privacy. ^b^Sites swabbed in three different location using a 10 × 10 cm template. ^c^Left and right shoes swabbed. ^d^Site could not be sampled.

### 2.2 Culture‐based analysis

The United States Department of Agriculture Food Safety and Inspection Service (USDA‐FSIS) laboratory guidebook for the isolation and identification of *Salmonella* from meat, poultry, pasteurized egg, and catfish products, and carcass and environmental sponges was followed up for culture‐based analysis (USDA/FSIS, 2014). Positive colonies were selected and one colony per plate was picked. In addition, culture‐based positive isolates were analyzed with a combination of biochemical assays: Lysine Iron Agar test (BD Difco, Sparks, MD) and Triple Sugar Iron Agar test (BD Difco). Positive colonies were further investigated in a slide agglutination assay using a *Salmonella* O antiserum polyvalent test for groups A through G+ iv following the manufacturer's instructions (BD Difco *Salmonella* O Antisera).

### 2.3 Molecular‐based analysis

Positive culture‐based samples were further analyzed by real‐time PCR. One colony from each agar plate was transferred directly and without any treatment to the PCR mixture. A protocol developed in our laboratory, that targets the invasion gene *inv*A present in all *Salmonella enterica* was followed (Bai et al., 2018). For every experiment, a nontemplate control, a non‐*Salmonella* control (*Escherichia coli* O157:H7 ATCC 43888) and four positive controls (*Salmonella* Newport from ATCC 6962, *Salmonella* Typhimurium from ATCC BAA‐215, *Salmonella* Typhimurium monophasic variant 4, [5], 12:i:‐ CA RM 17 305 obtained from USDA ARS Albany CA and *Salmonella* Typhimurium monophasic variant 4, [5],12:i:‐ NY FSL5‐580 obtained from the Department of Food Science at Cornell University) were added. A sample was considered PCR positive when the Ct value was lower than 40.

### 2.4 Statistical analysis

Samples exhibiting biochemical characteristics compatible with *Salmonella* were considered culture positives (C+) and samples that were serotyped as *S. enterica* by the molecular assay were named PCR positive (PCR+). Descriptive statistics were computed to depict the number and percentage of test positive samples by sampling site and feed mill type. Surfaces were denoted as positive if at least one of the three samples collected tested positive. Percent positive samples was calculated as the number of test positive samples divided by the total number of samples collected by sampling site and by feed mill. Associations between explanatory variables (sampling site and mill type) with the prevalence of positive samples were analyzed using generalized linear mixed models using Proc GLIMMIX in SAS 9.4 (SAS Institute Inc., Cary, NC). A binary distribution, logit link, Laplace approximation and a ridge‐stabilized Newton–Raphson algorithm were used. The outcome consisted of the presence of positive samples both by culture and molecular‐based analysis (dichotomous: positive vs. negative). Independent variables included: mill ID (each individual mill received an ID consisting of a number from 1 to 11), state (state where the mill is located) mill type (divided into mills producing mash or pelleted feed), and sample site (location within the mill that was analyzed). Depending on the fixed effect evaluated in the univariable and multivariable models, we incorporated a random intercept for feed mill nested within state (except when evaluating feed mill or state as fixed effect), to account for the hierarchical structure of the study. An initial univariable screen was followed by a multivariable model if more than one fixed effect was significant in the univariable screen. Mean probabilities and their 95% confidence intervals were computed and significance was indicated by *p* < 0.05.

## 3 RESULTS AND DISCUSSION

For this study, both C+ and PCR+ samples were considered: results from culture‐based analysis gave an indication of *Salmonella* genus presence (family of *Enterobacteriaceae*), while molecular‐based analysis provided specific information about *S. enterica* prevalence.

Samples that were not C+ were analyzed by biochemical tests (API 20E; Biomeriux, Durham, NC). Results indicated that the majority of these isolates were either *Enterobacter* or *Citrobacter*. Several studies have shown that *Enterobacteriaceae* counts tend to be higher in *Salmonella* positive samples and that the presence of Enterobacteriaceae can be considered as an indicator of hygiene in feed mill production systems and a tool to assess the likelihood of *Salmonella* incidence (Jones & Richardson, 2004). Nevertheless, since results from the Literature are conflicting, our discussion concentrated only on the presence of *Salmonella* (both C+ and PCR+) in feed mill environments.

Table 2 shows the outcome from the univariable model: mill ID (*p* < 0.001), state (*p* < 0.001) and sampling site (*p* = 0.0024) were significantly associated with the presence of *Salmonella* spp., while mill type (*p* = 0.3212) was not. Nevertheless, since most of the state selected for this study had only one feed mill visited, these two variable were considered confounded. The distribution of positive samples collected from feed mill facilities selected in this study is presented in Table 1. A total of 237 samples were tested: 157 (66.2%) resulted *Salmonella* C+ and 19.8% (*n* = 31) were also PCR+. All feed mills analyzed in this study had at least one C+ *Salmonella* site (Table 1). The percentage of C+ samples was greater in sampling sites corresponding to worker shoes (92.5%), finished product bin boot (81.8%), ingredient pit grating (80.6%), and floor dust in receiving area (80.6%). Conversely, fat intake inlet (20%), exterior pellet mill (33.3%) and finished feed (41.7%) showed the lowest percentage of positive samples in the analyzed facilities. In our study we also observed that overall the number of C+ samples decreased from the initial processing steps toward the finished product, following feed production flow. As highlighter in Figure 1 the manufacturing process within feed mill includes receiving, processing, storage‐packaging, loading, and delivery. Ingredient, people and cross‐contamination during production, load out, and delivery were all identified as potential hazard for microbial and viral introduction in feed mills (Cochrane et al., 2015). A biosecurity plan might offer an effective approach to reduce the likelihood of biological presence in feed mill manufacturing facilities, as well as microbial risk assessment and mitigation practices. Similar results of high pathogen presence in dust samples collected from manufacturing operations (33%–65%), storage areas (10%–27%), and worker shoes (9%–100%) were reported in a study that reviewed the practical measures to control *Salmonella* in animal feed (Jones & Richardson, 2004). This research highlighted the difficulty of detecting *Salmonella* in feed and the need to sample also dust and debris in feed manufacturing facilities to obtain a more sensitive indication of pathogen presence (Jones & Richardson, 2004). Based on these observations, in our study we selected sampling sites considering feed production flow, people traffic and dust accumulation. We also observed that the finished product bin boot had the highest number of C+ positive samples (81.1%) within the sampling sites in the production area. This equipment is in contact with the finished product before loading; therefore, it was identified as a high‐risk contamination point in our research: it might represent the suitable entry point for *Salmonella* in the feed to fork chain.

**Table 2:** Effects of variables on culture positive samples in the selected feed mills for this study

Variable	*M* (%)	95% CI	*p*‐Value
Mill ID			<0.001
1	27.3	10.3–55.3	
2	27.3	10.3–55.3	
3	37.1	15.6–65.2	
4	91.3	66.5–98.2	
5	88.7	64.2–97.2	
6	63.8	35.3–85.0	
7	76.2	38.4–94.3	
8	87.5	61.4–96.9	
9	33.1	13.1–61.9	
10	100	0–100	
11	79.2	51.3–93.2	
State			<0.001
Colorado	28.4	11.1–55.9	
Illinois	79.2	52.0–93.0	
Indiana	100	0–100	
Iowa	49.3	28.0–70.7	
Kansas	43.3	21.8–67.6	
Minnesota	87.4	61.8–96.8	
North Carolina	89.4	73.2–96.6	
Oklahoma	38.1	16.7–65.4	
Mill type			0.321
Mash	15.5	37.2–96.1	
Pelleted	20.3	19.1–85.5	
Sampling site			0.002
Ingredient pit grating	86.6	52.0–97.5	
Fat intake inlet	12.0	1.3–58.6	
Pellet mill	33.9	3.9–86.7	
Discharge bin boot	87.3	41.8–98.5	
Load‐out auger	39.5	7.8–83.4	
Finished feed	44.6	9.9–85.4	
Control room floor	67.7	27.7–92.0	
Receiving area floor	86.6	52.0–97.5	
Manufacturing area floor	63.2	24.0–90.4	
Warehouse area floor	53.7	17.6–86.2	
Worker shoes	97.5	75.0–99.8	
Broom	65.9	19.7–93.8	

Among the sampling sites that were not considered directly part of the production flow, worker shoes and broom had 95.2% and 63.6% C+ samples, respectively. These results highlight the high likelihood of microbial transfer and cross‐contamination within the facilities based on people movements (Cochrane et al., 2015). It was also observed that overall pelleted facilities had higher percentage of microbial presence as compared to mash mills in the final products (Table 1). The unfiltered air introduced into the system to cool the feed after the pelleting step might represent the source of recontamination in this type of facilities. Similar to our observations, also another study on *Salmonella* contamination in US swine feed reported higher pathogen presence in pelleted commercial feed product as compared to on‐farm mixed mash products (Davies et al., 2004).

All C+ positive samples were analyzed by qPCR and 31 (19.8%) were confirmed *S. enterica* (Table 1). Likewise, high pathogen presence was observed during the initial steps of production: ingredient pit grating (16.1%), floor dust in receiving area (16.1%), floor dust in manufacturing area (19.4%), floor dust in brake/control room (16.1%), exterior pellet mill (16.7%), and finished product bin boot (18.2%). No PCR+ samples were detected in load‐out auger and fat intake inlet. Finish feed showed 8.8% PCR+ samples. Within the sampling sites outside production flow, broom showed the greatest percentage of PCR+ (27.3%), followed by worker shoes (9.5%). As previously observed for C+ samples, microbial presence seems to be highly connected to people movement.

Since no data on weather condition during sampling were recorded and no biosecurity plan details were asked to the feed mill collaborators, the author think that a longitudinal study might be needed to better define the influence of mill location and seasonality on pathogen prevalence. At this point we can only hypothesize that the facilities where the highest amount of positive samples were detected did not have effective sanitation practices and/or cross‐contamination occurred from incoming ingredients, employees, trucks, or during other processing steps. Our results highlight the need of preharvest control measures in feed mill facilities both for human and animal foodborne pathogens. According to section 402 of the Federal Food, Drug, and Cosmetic Act (FD&C Act), FDA considers adulterated a feed that “is contaminated with a *Salmonella* serotype that is considered pathogenic to the animal intended to consume the animal feed and the animal feed will not subsequently undergo a commercial heat step or other commercial process that will kill the *Salmonella*.” For swine feed, only *Salmonella* Cholerasuis is considered as adulterant. This agent does not cause zoonotic disease. Nevertheless, certain animal serotypes, such as *Salmonella* enterica serovar *Typhimurium* and its monophasic variant serovar I 4,[5],12:i:‐, that are not considered animal feed adulterants at present, can be carried by pigs without clinical signs and might enter into the human food chain during postharvest operations (CDC, 2014).

Most peer‐reviewed studies on *Salmonella* presence in commercial feed manufacturing facilities focus on final product contamination, indicating the occurrence of pathogen infection, but they lack information regarding pathogen environmental presence (Jones, 2011; Li et al., 2012; Molla et al., 2010). Contaminated feed can represent a vehicle for *Salmonella* transmission to animals and therefore increase pathogen likelihood to be introduced into the human food (Crump & Griffin, 2002). Hence, understanding the mechanisms of contamination at preharvest level is instrumental for a more thorough hazard analysis and biosecurity plan development: the goal is to prevent and reduce pathogens contamination in animal feed and decrease the possible entrance into the human food chain (Houser et al., 2010; Li et al., 2012).

To our knowledge, this is the first study evaluating *Salmonella* presence in US feed mill environments. Our data showed that feed manufacturing facilities can represent a port of entry for the pathogen into the food supply chain and that effective mitigation strategies are needed to identify contamination sources and reduce risk. Future studies exploring the seasonality, genetic relatedness, as well as serotyping and antibiotic resistance profiles of *Salmonella* isolates are warranted to fully understand the epidemiology, ecology, and distribution of this pathogen in US feed mill environments.

## AUTHORS CONTRIBUTIONS

G.M. and V.T. carried out strain isolation. G.M. drafted the Manuscript, N.C. supervised statistical and epidemiological analysis and all the other authors contributed to experimental design, read, commented and approved the final manuscript.

## CONFLICT OF INTEREST

The authors confirm that this article content has no conflict of interest.

## Data Availability

All data are provided in full in the results section of this paper.
